# Item‐reduced Forgotten Joint Score provides adequate psychometric properties in ACLR patients

**DOI:** 10.1002/jeo2.12058

**Published:** 2024-06-11

**Authors:** Carolina Kekki, Tobias Wörner, Anders Stålman, Christoffer von Essen

**Affiliations:** ^1^ Department of Molecular Medicine and Surgery Karolinska Institute Stockholm Sweden; ^2^ Capio Artro Clinic, FIFA Medical Centre of Excellence, Sophiahemmet Hospital Stockholm Sweden

**Keywords:** ACL, ACL reconstruction, Forgotten Joint Score, patient‐reported outcome, validation

## Abstract

**Purpose:**

The purpose of this study was to evaluate content validity, test–retest reliability, internal consistency, construct validity, responsiveness and floor/ceiling effects of Forgotten Joint Score (FJS) for assessing functions in activities of daily living (ADL) following anterior cruciate ligament reconstruction (ACLR) and perform an item reduction of FJS.

**Methods:**

Swedish‐speaking ACLR patients in one surgical clinic were eligible. Content validity was evaluated through patient responses and patient and expert clinician relevance ratings, omitting items with low relevance. Principal component factor analysis, Cronbach's *⍺*, paired *t* test, correlations between FJS and Knee Injury and Osteoarthritis Outcome Score (KOOS), Cohen's *d* effect sizes (ESs) and standardized response mean (SRM) were used to evaluate internal consistency, test–retest reliability, construct validity and responsiveness. Floor/ceiling effects were calculated. FJS was expected to reveal one dominant factor, a Cronbach's *⍺* between 0.70 and 0.95, correlations >0.50 to all KOOS subscores, highest for ADL, moderate ES and SRM, floor/ceiling effects <15%.

**Results:**

One hundred and seventy‐six participants (103 for assessing internal consistency, construct validity, responsiveness and floor/ceiling effects; 73 for assessing test–retest reliability and content validity) were included. Item reduction yielded a nine‐item FJS (FJS‐9). FJS‐12 and FJS‐9 demonstrated sufficient content validity (95% confidence interval [CI], 2.5–3.1, respectively, 2.9–3.3). FJS‐9 was found unidimensional, and FJS‐12 was multidimensional. Cronbach's *⍺* was 0.94 for FJS, and the intraclass correlation coefficient > 0.90. FJS showed significant correlations >0.65 to all KOOS subscales, moderate ES and SRM > 0.50. No floor/ceiling effects were found.

**Conclusion:**

FJS‐9 demonstrated adequate validity for the evaluation of joint awareness in ACLR patients and can be a valuable tool to assess ADL and joint awareness.

**Level of Eidence:**

Level III.

**Abbreviations**
ACL: anterior cruciate ligament
ACLR: anterior cruciate ligament reconstruction
ADL: activities of daily living
CI: confidence interval
COSMIN: Consensus‐based Standards for the selection of health status Measurement Instruments
ES: effect size, Cohen's *d*
FJS: Forgotten Joint Score
ICC: intraclass correlation coefficient
IQR: interquartile range
KOOS: Knee Injury and Osteoarthritis Outcome Score
MDC: minimal detectable change
PROM: patient‐reported outcome
QOL: quality of life
*r*
: Pearson's correlation coefficient
SD: standard deviation
SEM: standard error of measurement
Sport: sport or recreation function
SRM: standardised response mean

## BACKGROUND

Anterior cruciate ligament reconstruction (ACLR) is one of the most common surgical procedures in modern orthopaedic surgery. The incidence of ACL injury in Sweden is about 80 per 100,000 individuals [30] with a mean age of 27 years for women and 28 years for men [30]. In Sweden, approximately 50% of the ACL injuries are treated surgically [30], with the goal of restoring the stability and function of the knee. Patient‐reported outcomes (PROMs) provide a patient‐centred perspective of outcomes such as symptoms, function and quality of life [12] and are, therefore, essential for the evaluation of orthopaedic treatments. PROMs should be reliable, valid and sensitive to ensure adequate evaluation of postoperative knee function [5]. In Sweden, the Knee Injury and Osteoarthritis Outcome Score (KOOS) is the most frequently used PROM to evaluate ACLR patients [30]. Since ACLR patients generally perform well, it is important that a PROM used in this patient group can discern small differences in outcomes. KOOS has several issues, one being a low content validity [9, 17] and another one being a ceiling effect. Content validity evaluates how well an instrument covers all relevant parts of the construct it aims to measure. If a PROM has a low content validity, it may not be optimal to use in a certain patient group. However, since KOOS is the most frequently used PROM in ACLR patients in Sweden today [30], it is the most important PROM to compare with when validating a PROM for ACLR patients. A ceiling effect occurs when many patients cluster near the best possible score, therefore, reducing the PROMs ability to measure the outcome above a certain level. KOOS has been demonstrated to suffer from such a ceiling effect, which is mainly evident in the subscale ‘activities of daily living’ (‘ADL’) and ‘pain’ [6, 36]. New PROMs are needed to evaluate these constructs more accurately.

In 2012, Behrend et al. [3] introduced a new PROM called the Forgotten Joint Score‐12 (FJS‐12). FJS‐12 was developed through a combination of literature research and expert opinion and includes 12 items assessing awareness of the affected joint during various ADL activities on a five‐point Likert scale [0p (never) to 4p (mostly)] [3]. Joint awareness encompasses all unintended perceptions related to a joint, such as pain, stiffness and instability, but also more subtle sensations such as subjective dysfunction and discomfort [5]. Since healthy joints typically go unnoticed in everyday life, assessment of joint awareness appears relevant in the follow‐up of surgical treatment of joints [3]. FJS‐12 has been validated for patients following knee and hip arthroplasty through its ability to discriminate well between good, very good and excellent outcomes [3, 7, 24, 26]. Recent studies from Germany and United States have also validated FJS‐12 in the assessment of ADL in patients following ACLR [4, 32]. Even though FJS‐12 has shown to be a reliable tool with adequate construct validity in the assessment of ADL function of ACLR patients, content validity of the scale has not been evaluated in this group of patients. The Consensus‐based Standards for the selection of health status Measurement Instruments (COSMIN) guidelines consider content validity to be the most important psychometric property of a PROM and recommend it to be evaluated by involving expert clinicians and patients [22]. Furthermore, no item reduction of FJS‐12 has previously been performed in ACLR patients. Low response rates are a common problem with PROMs [23], and by asking patients as few yet relevant questions as possible, the motivation to respond could increase.

The aim of the current study was to evaluate the content validity, test–retest reliability, internal consistency, construct validity, responsiveness and floor/ceiling effects of FJS as an outcome measure for ACLR using the Swedish translation of FJS. Additionally, an item reduction was performed to further adapt the scale to the target population. It was expected that FJS would demonstrate adequate content validity, good test–retest reliability, high internal consistency, adequate construct validity, responsiveness to change and minimal floor and ceiling effects.

## METHODS

### Study design

All Swedish‐speaking patients undergoing ACLR between November 2020 and November 2021 (patient group 1) or between January and May 2019 (patient group 2) in a single surgical clinic specialized in arthroscopic procedures were eligible for inclusion in this psychometric study. Patient group 1 was used to evaluate internal consistency, construct validity, responsiveness and floor/ceiling effects. Patient group 2 was specifically recruited to evaluate test–retest reliability and content validity, as the required data for these analyses were not available in patient group 1. In accordance with Behrend et al. [3], patients were excluded if more than four FJS‐12 items were missing in FJS‐12 or/and if the KOOS data provided by The Swedish Knee Ligament Registry was incomplete. To ensure the methodological quality of the study, the COSMIN guidelines were used to plan this study and reporting its results [18]. Ethical approval for this study was obtained from the regional ethics committee (2016/1613‐31/32).

### Participants and data collection

Patient group 1 was asked to complete the KOOS and FJS‐12 questionnaires via a web survey during an information meeting prior to surgery. One year following surgery, these patients received the same questionnaires via postal mail and sent their responses back to the clinic. Patient group 2, consisting of patients 4 years following ACLR, was contacted by phone in May and June 2023 and provided with a web survey with FJS‐12 by email. These patients received FJS‐12 twice, with an interval of at least 2 weeks between send outs. It was assumed that patients had reached a stable state in the construct measured by FJS‐12 4 years following ACLR, which is crucial for the evaluation of test–retest reliability. Furthermore, a 2‐week interval was considered long enough for participants not to recall their first responses to the questionnaire. Patient group 2 was also asked to rate the relevance of each FJS‐12 item. Additionally, a group of clinicians, including expert surgeons and physiotherapists, was recruited in May and June 2023 and asked to rate the relevance of each FJS‐12 item. Data on KOOS, sex and age at surgery were retrieved from the Swedish Knee Ligament Registry.

### Surgical procedure

All patients underwent surgery using a single‐bundle autologous quadruple hamstring tendon, quadruple tendon with or without a bone block or bone‐patellar tendon‐bone technique. The graft was chosen according to the surgeon's preference. The femoral tunnel was drilled using an anteromedial portal technique. In the majority of cases, the grafts were fixed using a TightRope device (Arthrex) and No. 2 Ethibond sutures (Ethicon) tied over an bicortical screw with a washer as a post on the tibial side. Meniscal repair was performed when needed, using an all‐inside technique. All the patients followed a standardized postoperative rehabilitation protocol. In the event of an isolated ACLR or an ACLR with concomitant meniscus resection, full weight bearing and full range of motion were encouraged as tolerated. If meniscal repair was performed, patients wore a postoperative hinged knee brace for 6 weeks. Flexion was limited from 0° to 30° for the first 2 weeks after surgery, from 0° to 60° for the third and fourth weeks and from 0° to 90° for the 5th and 6th weeks.

### FJS‐12

FJS‐12 consists of 12 items related to joint awareness during activities of everyday life. Each item is answered on a five‐point Likert scale with the following response options: *never* (0 p); *almost never* (1 p); *seldom* (2 p); *sometimes* (3 p) and *mostly* (4 p). The initial raw scores were then converted to a scale ranging from 0 to 100, with a higher score indicating better outcome [3]. The conversion was conducted by dividing the summarized score by the number of completed items, multiplying the result by 25 and thereafter subtracting it from 100. The Swedish‐language version of FJS‐12 was provided by the developers and translated in accordance with the report ‘Principles of Good Practice for the Translation and Cultural Adaption Process for Patient Reported Outcomes (PRO) Measures’ [34].

### KOOS

KOOS consists of 42 items, assessing knee function across five subscales: pain, symptoms, ADL, sport or recreation function (sport) and knee‐related quality of life (QOL). Each item is answered on a five‐point Likert scale. The items of each subscale were then transformed to a subscale score ranging from 0 to 100, with a higher score indicating better outcome [25].

### Sample size estimation

Sample sizes were determined based on the COSMIN study design criteria for PROMs [18]. The COSMIN guidelines recommend ≥100 patients for a ‘very good’ rating or 50–99 patients for an ‘adequate’ rating on test–retest reliability, ≥100 patients for a ‘very good’ rating on internal consistency and on construct validity, ≥50 patients for a ‘very good’ rating on content validity, ≥7 expert clinicians for a ‘very good’ rating on content validity and ≥100 patients for a ‘very good’ rating on responsiveness. We aimed to include 100 patients for the evaluation of internal consistency, construct validity, responsiveness and floor/ceiling effects (patient group 1), 50 patients for the evaluation of test–retest reliability and content validity (patient group 2) and seven expert clinicians for the evaluation of content validity.

### Assessment of psychometric properties

#### Item reduction

Items were retained if they met at least two of the following criteria: (a) The responses from patients showed central tendencies close to the midpoint of the possible range with a large spread (i.e., no floor/ceiling effects) and/or the item demonstrated high relevance based on (b) patient and expert rating, with a mean relevance score of at least 67% of the maximum score (equivalent to ≥ 2.7) and/or (c) at least 67% of all patients and experts rating the item as ‘somewhat relevant’ [33, 35].

#### Content validity

Content validity was assessed by asking patients and expert clinicians to rate each FJS‐12 item on a scale from 1 to 4 (1 = *not relevant*, 2 = *somewhat relevant*, 3 = *quite relevant*, 4 = *very relevant*). The expert clinicians consisted of orthopaedic surgeons and physiotherapists specialized in the treatment of ACL injuries. Mean relevance scores were calculated for each item as well as for the whole scale. Sufficient content validity was assumed for relevance scores of at least 2.7 or greater with at least 67% of patients rating the item as at least ‘somewhat relevant’ [33, 35].

#### Internal consistency

Internal consistency was assessed by conducting an exploratory principal component factor analysis and by calculating Cronbach's *⍺*. Exploratory principal component analysis identifies underlying constructs (factors) within data by examining correlations among variables. Factors are associated with loadings indicating the strength and direction of their relationship with the variables. Factor analysis determines the number of factors explaining the most variance in the data, guided by eigenvalues. According to Kaiser's criterion, factors with eigenvalues > 1 are retained [28]. It was expected for the factor analysis to reveal one dominant factor with an eigenvalue exceeding one [28]. Cronbach's *⍺* was expected to be between 0.70 and 0.95, which is considered adequate according to Terwee et al. [29].

#### Test–retest reliability

Test–retest reliability was expressed by intraclass correlation coefficient (ICC), and sufficient test–retest reliability was assumed for an ICC > 0.70 as recommended as the minimum standard for reliability by Terwee et al. [29]. Hypothesis testing was done by a paired *t* test, only including patients from Patient group 2 who reported no difference in their knee joint between the first and second administration of the questionnaire. By only including patients who did not report a difference in their knee joint, it is possible to evaluate the consistency and reproducibility of FJS without potential confounding effects from changes in knee joint status.

#### Construct validity

Convergent construct validity was tested by correlating FJS to KOOS using Pearson's correlation coefficient (*r*). Based on previous literature [4], evidence of convergent construct validity was assumed for positive correlations of at least 0.50 to the KOOS domains pain, symptoms, sport and QOL. The correlation was expected to be higher for the ADL domain than for the other subscales since ADL is the construct that FJS is developed to measure.

#### Responsiveness

Responsiveness was evaluated by calculating Cohen's *d* as a measure of effect size (ES) and by calculating the standardized response mean (SRM). Small effects were considered >0.20, moderate effects were considered >0.50 and large effects were considered >0.80 [11]. FJS was expected to have a moderate ES and SRM.

#### Floor and ceiling effects

Potential floor and ceiling effects were evaluated by calculating the percentage of participants with the lowest or highest possible score. In accordance with quality criteria proposed by Terwee et al. [29], floor and ceiling effects >15% were determined as pronounced, and FJS was expected to have floor and ceiling effects <15%.

### Statistical analysis

All statistical analyses were performed using Rstudio version 4.3.1. Score distributions were reported as mean values and standard deviations (SDs) for normally distributed data, while median and interquartile range were presented for nonnormally distributed data. Normal distribution of data was judged by visual inspection. Exploratory principal component factor analysis and Cronbach's *⍺* were used to assess internal consistency. Test–retest reliability was reported with a 95% confidence interval (CI). To assess test–retest reliability, the ICC was calculated for a two‐way random effect model with measures of absolute agreement. The standard error of measurement (SEM) and minimal detectable change (MDC) were used as parameters of measurement error. The ICC was calculated for patient group 2 and subsequently classified as poor (0–0.50), moderate (0.50–0.75), good (0.75–0.90) or excellent (0.90–1.00) [13]. Convergent construct validity was reported with a 95% CI. Pearson's correlation coefficients (*r*) were used to assess correlation between FJS and KOOS. Correlations between FJS and each of the KOOS domains were classified as negligible (0–0.10), weak (0.10–0.39), moderate (0.40–0.69), strong (0.70–0.89) or very strong correlation (0.90–1.00) [27]. The content validity of FJS was assessed by relevance ratings, and floor and ceiling effects were assessed for FJS.

## RESULTS

**Figure 1:**
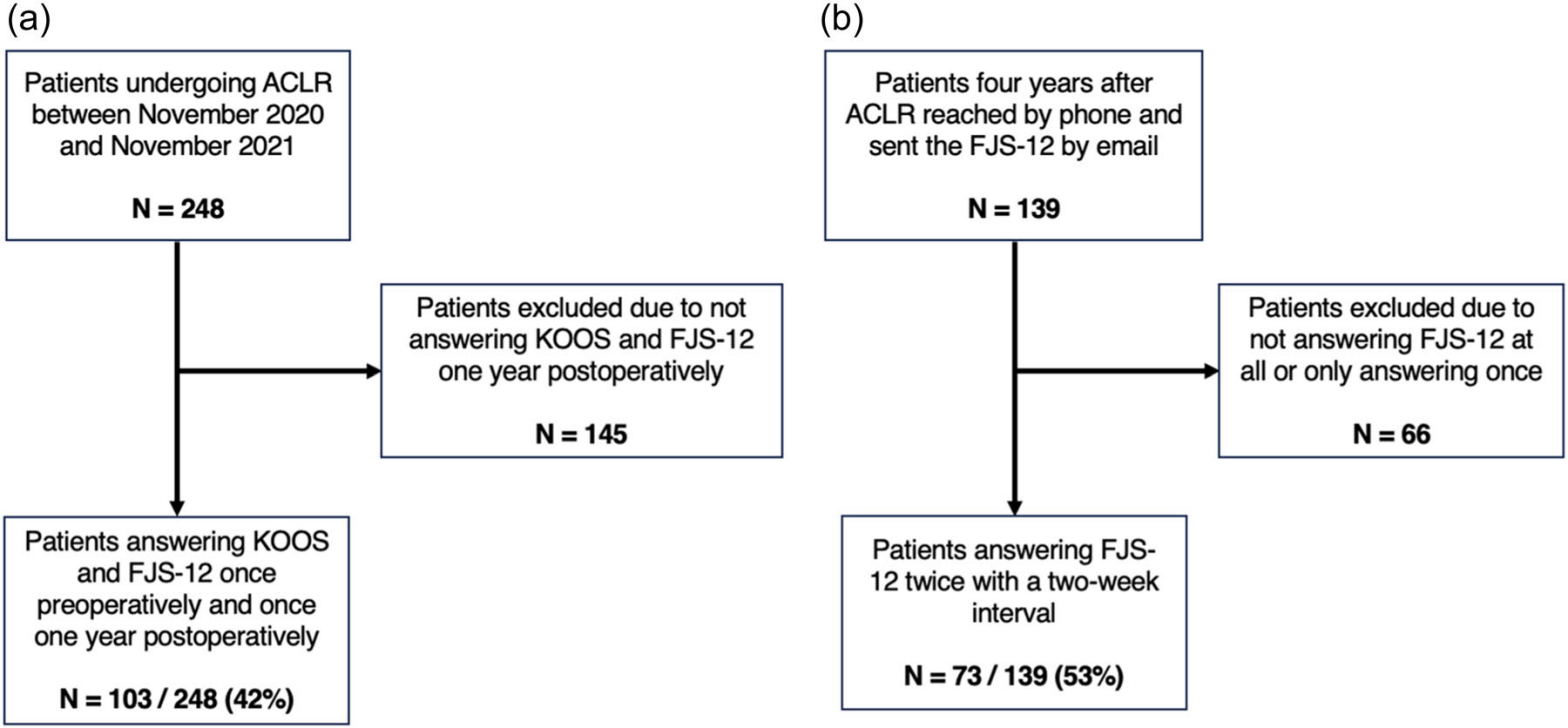
Patient flow into the study for patient group 1 (a) and patient group 2 (b). ACLR, anterior cruciate ligament reconstruction; FJS, Forgotten Joint Score; KOOS, Knee Injury and Osteoarthritis Outcome Score.

The final sample consisted of 103 patients for the evaluation of internal consistency, construct validity, responsiveness and floor/ceiling effects (Patient group 1) and 73 patients for the evaluation of content validity and test–retest reliability (Patient group 2). A total of 145 patients were excluded from patient group 1 due to not answering KOOS and FJS‐12 1 year postoperatively, and 66 patients from patient group 2 were excluded either for not answering FJS‐12 at all or for responding only once. The excluded patients shared similar characteristics to the included patients, with a mean age of 32 years (SD = 11) and 53.8% being females. For the evaluation of content validity, 37 expert clinicians (12 surgeons, 25 physiotherapists) were also included. Patient flow into the study is illustrated in Figure 1, and patient demographics are described in Table 1.

**Table 1:** Baseline demographic characteristics of the two study groups.

Characteritics	Patient group 1 (*n* = 103)	Patient group 2 (*n* = 73)
Age in years [mean (SD)]	33 (12)	31 (11)
Gender		
Male [% (*n*)]	45.6% (47)	42.5% (31)
Female [% (*n*)]	54.4% (56)	57.5% (42)

Abbreviation: SD, standard deviation.

### Item reduction and content validity

Item reduction based on relevant scores and patient scores yielded nine items with adequate content validity. Item 1 (Are you aware of your knee joint in bed at night?), 4 (Are you aware of your knee joint when you are taking a bath/shower?) and 5 (Are you aware of your knee joint when you are traveling in a car?) were omitted from the scale. The original FJS‐12 as a total scale received a mean relevance score of 2.8 (95% CI, 2.5–3.1); the item reduced (from now on: FJS‐9) received a mean relevance score of 3.1 (95% CI, 2.9–3.3). Eighty‐five patients from patient group 2 were included in the relevance rating, which was included in the first administrations of the questionnaire, and 37 experts also rated the relevance of FJS‐12. Patient scores and relevance ratings alongside the rational for excluding items can be found in Table 2.

**Table 2:** Descriptive data for FJS‐12 1 year postoperatively and relevance rating by patients and experts.

Item	Patient scores (*n* = 103)		Relevance rating (*n *= 122)	
Are you aware of your knee joint…	Mean (SD)	Median (IQR)	Mean (SD)	Relevant (%)
1. …in bed at night?	1.0 (1.4)	0 [0, 1.0]	2.1 (1.0)	68.8
2. …when you are sitting on a chair for more than 1 h?	1.7 (1.6)	1.0 [0, 3.0]	2.7 (1.0)	84.5
3. …when you are walking more than 15 min?	1.2 (1.4)	1.0 [0, 2.0]	2.9 (1.1)	84.4
4. …when you are taking a bath/shower?	0.5 (0.8)	0 [0, 1.0]	1.9 (1.0)	57.3
5. …when you are traveling in a car?	1.2 (1.2)	1.0 [0, 2.0]	2.4 (1.0)	77.9
6. …when you are climbing stairs?	1.5 (1.3)	1.0 [0, 2.0]	3.1 (1.0)	88.5
7. …when you are walking on uneven ground?	1.7 (1.4)	2.0 [0.5, 3.0]	3.1 (1.1)	88.6
8. …when you are standing up from a low‐sitting position?	2.2 (1.4)	2.0 [1.0, 3.0]	3.3 (0.9)	93.5
9. …when you are standing for long periods of time?	1.6 (1.5)	1.0 [0, 3.0]	3.0 (1.0)	89.3
10. …when you are doing housework or gardening?	1.3 (1.4)	1.0 [0, 2.0]	2.8 (1.0)	86.9
11. …when you are taking a walk/hiking?	1.8 (1.5)	2.0 [0, 3.0]	3.1 (1.0)	89.3
12. …when you are doing your favourite sport?	2.8 (1.3)	3.0 [2.0, 4.0]	3.6 (0.7)	98.4

Abbreviations: FJS, Forgotten Joint Score; IQR, interquartile range; SD, standard deviation.

### Internal consistency

The factor analysis revealed two dominant factors for FJS‐12 and one for FJS‐9 (Table 3). Regarding FJS‐12, factor 1 exhibited strong positive loadings (≥0.66) on all items. Additionally, items 1, 2, 5 and 8 displayed positive factor loadings on factor 2, with loadings of 0.29, 0.67, 0.58 and 0.18, respectively. Cronbach's *⍺* for both FJS‐12 and FJS‐9 was 0.94, which can be considered adequate internal consistency. Factor loadings for all items of FJS‐12 and FJS‐9 onto factors 1 and 2 are presented in Table 4.

**Table 3:** Eigenvalues, factor variance and Cronbach's *⍺* of FJS‐12 and FJS‐9 1 year postoperatively.

		Eigenvalue				
	*n*	Factor 1	Factor 2	First factor variance (%)	Second factor variance (%)	Cronbach's *⍺*
FJS‐12	103	7.5	1.2	62.5	10.3	0.94
FJS‐9	103	6.1	0.8	67.8	9.0	0.94

Abbreviation: FJS, Forgotten Joint Score.

**Table 4:** Factor loadings onto factor 1 and factor 2 for all items of FJS‐12 and FJS‐9.

	FJS‐12		FJS‐9	
Item	Factor 1	Factor 2	Factor 1	Factor 2
1	0.75	0.29	‐	‐
2	0.66	0.67	0.60	0.71
3	0.87	−0.06	0.88	0.04
4	0.68	−0.29	‐	‐
5	0.69	0.58	‐	‐
6	0.83	−0.02	0.85	0.08
7	0.88	−0.20	0.89	−0.17
8	0.71	0.18	0.71	0.34
9	0.84	−0.24	0.86	−0.18
10	0.89	−0.21	0.89	−0.15
11	0.88	−0.32	0.90	−0.27
12	0.75	−0.09	0.77	−0.11

*Note*: Items excluded from FJS‐9 are denoted by ‘‐’.

Abbreviation: FJS, Forgotten Joint Score.

### Test–retest reliability

To assess test–retest reliability, 60 patients were included. Both FJS‐12 and FJS‐9 had excellent test–retest reliability despite significant differences between test and retest scores. ICCs, group differences, SEM and MDC are summarized in Table 5.

**Table 5:** Test–retest reliability analysis of FJS 4 years postoperatively.

	*n* = 60									
	Mean 1 (SD)	Mean 2 (SD)	Individual ICC (95% CI)	Group ICC (95% CI)	Individual SEM	Group SEM	Individual MDC	Group MDC	Mean difference (95% CI)	*p* Value
FJS‐12	62.5 (26.9)	57.5 (27.4)	0.92 (0.83–0.96)	0.96 (0.91–0.98)	7.36	5.31	1.34	0.97	5.07 (2.64–7.49)	<0.001
FJS‐9	57.6 (29.7)	52.9 (29.8)	0.93 (0.86–0.96)	0.96 (0.93–0.98)	7.79	5.61	1.42	1.02	4.70 (2.08–7.32)	<0.001

Abbreviations: CI, confidence interval; FJS, Forgotten Joint Score; ICC, intraclass correlation coefficient; MDC, minimal detectable change; SD, standard deviation; SEM, standard error of measurement.

### Construct validity

In accordance with our expectations, strong correlations were found between FJS‐12, FJS‐9 and all KOOS subscales (Table 6). Neither FJS‐12 nor FJS‐9 showed a significantly higher correlation with the KOOS subscale ADL compared to other subscales.

**Table 6:** Correlation of FJS‐12 and FJS‐9 with KOOS 1 year postoperatively.

*n* = 103		
KOOS subscale	Rho (95% CI)	*p* Value
FJS‐12		
Pain	0.77 (0.68–0.84)	<0.001
Symptoms	0.69 (0.58–0.78)	<0.001
ADL	0.77 (0.67–0.84)	<0.001
QOL	0.77 (0.68–0.84)	<0.001
Sport	0.71 (0.60–0.79)	<0.001
FJS‐9		
Pain	0.76 (0.66–0.83)	<0.001
Symptoms	0.66 (0.53–0.76)	<0.001
ADL	0.72 (0.61–0.80)	<0.001
QOL	0.78 (0.69–0.85)	<0.001
Sport	0.72 (0.61–0.80)	<0.001

Abbreviations: ADL, activities of daily living; CI, confidence interval; FJS, Forgotten Joint Score; KOOS, Knee Injury and Osteoarthritis Outcome Score; QOL, quality of life.

### Responsiveness

Both the ES and SRM for FJS were found moderate. Cohen's *d* (ES) was 0.66 for FJS‐12 and 0.69 for FJS‐9, while SRM was 0.59 for FJS‐12 and 0.58 for FJS‐9.

### Floor and ceiling effects

No pronounced floor or ceiling effects were observed for either FJS‐12 or FJS‐9. Both FJS‐12 and FJS‐9 had a floor effect of 1.0% and a ceiling effect of 1.0%. No pronounced floor effect was observed for KOOS. Pronounced ceiling effects were observed for KOOS in the subscales pain and ADL. The floor and ceiling effects of KOOS, FJS‐12 and FJS‐9 are presented in Table 7.

**Table 7:** Floor and ceiling effect for KOOS subscales, FJS‐12 and FJS‐9 1 year postoperatively.

Scale	Floor effect (%)	Ceiling effect (%)
FJS‐12	0.97	0.97
FJS‐9	0.97	0.97
KOOS		
Pain	0.00	15.53
Symptom	0.00	4.85
ADL	0.00	35.92
QOL	0.97	7.77
Sport	0.00	1.94

Abbreviations: ADL, activities of daily living; FJS, Forgotten Joint Score; KOOS, Knee Injury and Osteoarthritis Outcome Score; QOL, quality of life.

## DISCUSSION

In the current study, psychometric properties of FJS were assessed as an outcome measure for ACLR, and an item reduction based on patient responses and both patient and expert ratings was performed. The item‐reduced version FJS‐9 was found to be an internally consistent, unidimensional and valid tool for the evaluation of joint awareness after ACLR.

The current study found FJS to have adequate content validity, indicated by a mean relevance score of 2.8 (95% CI, 2.5–3.1) for FJS‐12 and 3.1 (95% CI, 2.9–3.3) for FJS‐9. Common problems of PROMs are the low response rates [23], and a concise score with items that are highly relevant to the patients increases the likelihood of patients responding to it. FJS‐9 is both concise and, as showed in the current study, relevant to ACLR patients.

The factor analysis found FJS‐9 to be unidimensional, while FJS‐12 was found multidimensional. Positive factor loadings onto a second factor were observed in four of the FJS‐12 items, accounting for 10.3% of the total variance. Both the original validation of FJS‐12 for ACLR [4] and studies on knee and hip arthroplasty [2, 8] have previously found FJS‐12 to be unidimensional. The multidimensionality found in the current study may, in part, be attributed to the use of the Swedish version of FJS‐12, which has not been explored regarding its factorial structure. Linguistic variations, along with differences in cultural and healthcare contexts, could contribute to variations in responses. Furthermore, the factor analysis was conducted using data from patients 1 year after ACLR, and the relatively short timeframe may have contributed to the multidimensionality. The items loading onto the second factor appeared to share a common characteristic related to more passive ADL including laying down in bed, sitting for more than an hour and traveling by car. When reducing the scale, two out of the four items loading into the second factor were omitted. Considering that joint awareness is often less pronounced during periods of inactivity [16], it is reasonable that a majority of the omitted items loaded onto the second factor. Consequently, FJS‐9 demonstrates greater relevance to ACLR patients compared to FJS‐12.

The internal consistency and test–retest reliability were found to be adequate for FJS. Both FJS‐12 and FJS‐9 exhibited a high Cronbach's *⍺* of 0.94, consistent with prior research evaluating the internal consistency of FJS‐12 in ACLR populations [4, 14, 32]. The test–retest reliability analysis showed an ICC > 0.90 at the individual level and 0.96 at the group level for both FJS‐12 and FJS‐9. At the group level, this is similar to findings of a systematic review by Adriani et al. [1] including 13 studies on FJS‐12 both for knee and hip arthroplasty, reporting ICC values exceeding 0.8 across all studies. KOOS has previously shown a high internal consistency and test–retest reliability for evaluation of ACLR, comparable to the results of the current study. Previous studies have found a Cronbach's *⍺* ranging from 0.79 to 0.97 in all subscales [19, 20, 21] and an ICC > 0.8 in all subscales [19, 20, 21]. Hence, FJS demonstrates adequate internal consistency, similar to KOOS, as assessed by COSMIN, and meets the minimum standard for test–retest reliability set by Terwee et al. [29].

Measurement error was evaluated both on the group level and individual level. SEM assesses the measurement error of a test, while MDC can be regarded as a threshold for identification of minimal clinical change exceeding the measurement error. In the current study, a notably lower individual MDC was found for FJS‐12 (1.34) and FJS‐9 (1.42) compared to a recent study by Longo et al. [15] on FJS‐12 and knee arthroplasty reporting an individual MDC value of 9.9. However, the follow‐up for the study by Longo et al. was only 6 months, which may affect the MDC as patients must be clinically stable for this measure to be evaluated correctly. In the current study, the follow‐up was 4 years. Additionally, there is a significant difference in patient demographics between these two kinds of surgeries, with the patients in Longo et al.'s study having a mean age of 74 years. To date, no studies have evaluated the test–retest reliability of FJS‐12 specifically for ACLR. Hence, FJS holds potential not only for research purposes but also for evaluating changes in joint awareness of individual patients in clinical settings. Construct validity showed, in accordance with our expectations, correlations exceeding 0.50 between FJS‐12 and FJS‐9 and the KOOS subscales. However, in contrary to our expectations, the correlation between FJS and the KOOS subscale ADL was not found to be higher than that observed for the other subscales. An explanation to this could be the high ceiling effect of KOOS [6, 36], giving it a skewed distribution towards the upper ranges of the score that both FJS‐12 and FJS‐9 are lacking.

Regarding responsiveness, both FJS‐12 and FJS‐9 showed a moderate ES and SRM > 0.6 in accordance with our expectations. A high ES of 1.0 was found in patients with knee arthroplasty assessed from preoperative to six months postoperative [15], while a larger ES (3.6) and SRM (1.6) were found in patients with hip arthroplasty assessed from preoperatively to 1 year postoperatively [31]. Aside from these two studies, no other studies on FJS‐12 have evaluated ES and SRM from preoperatively to postoperatively. Patients undergoing ACLR are younger, healthier and expect a higher functionality of their knee joint post‐ACLR compared to patients undergoing arthroplasty. Therefore, it is logical that the ES of arthroplasty patients, measured from preoperatively to postoperatively, is higher than that of FJS.

As expected, FJS demonstrated a low ceiling effect. A previous study on FJS‐12 and ACLR by Lee et al. [14] found a ceiling effect of 8.7%, while Behrend et al. [4] found ceiling effects of 12.1% at a mean follow‐up of 2.6 years and 15.5% at a mean follow‐up of 11.6 years. A third study by Vermeijden et al. [32] observed a high ceiling effect of 21.9%. However, this study included patients undergoing ACLR between 2008 and 2018, resulting in potential follow‐up periods of up to 12 years. High ceiling effects were also observed when compared with other PROMs. In the current study, no floor effect was observed for FJS, which is consistent with previous studies [15, 32]. In contrast to FJS, KOOS have previously shown a large ceiling effect, mainly in the subscales pain and ADL [6, 36]. This is in line with the results of the current study, where pronounced ceiling effects of 15.5%, respectively, 35.9% were observed for KOOS in the subscales pain and ADL. When scores cluster at the top of the scale, it becomes impossible to differentiate between patients with good and excellent outcomes. Since ACLR patients typically are younger, healthier and more active than many other patient groups undergoing orthopedic surgery, there is an increased demand within this specific patient group to distinguish between good and excellent outcomes. KOOS is not well‐suited for this, due to its high ceiling effect.

### Clinical applications

The concise nature of FJS makes it suitable for regular monitoring of post‐ACLR patients, allowing easy integration into clinical workflows and enabling early detection of functional issues related to ADL. The low individual MDC values indicate that FJS holds potential not only for research purposes but also for evaluating changes in joint awareness of individual patients in clinical settings. FJS could serve as a valuable complement to KOOS, especially in this patient group since ACLR‐patients generally perform well postoperatively.

### Methodological considerations

The current study is the first to evaluate the content validity and test–retest reliability of FJS in ACLR patients, and the first study to do a scale reduction of FJS‐12 in this patient group. Additionally, a factor analysis was conducted which has only been done in a few previous studies on FJS. The current study followed COSMIN guidelines to ensure methodological quality and had decent sample sizes resulting in an ‘adequate’ or ‘very good’ rating on sample sizes across all measures.

There are also a few methodological considerations in the current study. There was a considerable number of missing responses regarding KOOS and FJS‐12, with a response rate of 40%–50%. Low response rates are a common issue regarding PROMs [30]. However, findings in a study by Ingelsrud et al. [10], using data from the Norwegian Knee Ligament Registry, indicated that patients who did not respond to KOOS postoperatively did not significantly differ from those who did respond. Hence, it is less probable that the response rate had a substantial effect on the results of the current study.

ES and SRM were used to evaluate responsiveness instead of an anchor‐based method. COSMIN recommend using either the smallest detectable change and relating it to the minimal important change (MIC) or measuring the area under the receiver operating characteristics (ROC) curve (AUC) [29]. However, to calculate the MIC, the global rating of change is needed, and such data was not available. ROC and AUC rely on an external criterion and are more challenging to interpret. Furthermore, the current study developed a new short‐form survey using existing FJS‐12 scores rather than validating it in a new cohort of patients. No patients were asked to complete FJS‐12 and FJS‐9 separately for a direct comparison. Given that the order of items may influence responses, it is possible that the items of FJS‐9 would have been answered differently if encountered on their own. The advantage of this method is that, as the validated FJS‐9 was derived from FJS‐12, it can be calculated for all previously administered FJS‐12 surveys for comparison. However, future studies are needed to validate FJS‐9 in a separate cohort to confirm its validity.

## CONCLUSION

The item‐reduced version of FJS‐12 (FJS‐9) demonstrated adequate validity for the assessment of joint awareness in ACLR patients. FJS‐9 demonstrated psychometric properties that has potential to be as strong as those of FJS‐12. FJS is better than KOOS at discriminating between good and excellent outcomes and showed a greater ability of evaluating ADL in individual patients, empathizing that FJS‐9 can be a valuable tool and complement to KOOS in assessing ADL and joint awareness.

## AUTHOR CONTRIBUTIONS

Tobias Wörner, Anders Stålman and Christoffer von Essen contributed to the development of the research questions and study design. Carolina Kekki conducted the data analysis and drafted the manuscript. Christoffer von Essen, Tobias Wörner and Carolina Kekki interpreted the results of the data analysis. Tobias Wörner, Anders Stålman and Christoffer von Essen revised and approved the manuscript. All authors read and approved the final manuscript.

## CONFLICT OF INTEREST STATEMENT

The authors declare no conflict of interest.

## ETHICS STATEMENT

Ethical approval for this study was obtained from the regional ethics committee (2016/1613‐31/32). Informed consent was obtained by each participant.

## Data Availability

All data and materials are available on reasonable request to the corresponding author.
